# Changes in Loss Sensitivity During Treatment in Concurrent Disorders Inpatients: A Computational Model Approach to Assessing Risky Decision-Making

**DOI:** 10.3389/fpsyt.2021.794014

**Published:** 2022-01-28

**Authors:** Stefanie Todesco, Thomas Chao, Laura Schmid, Karina A. Thiessen, Christian G. Schütz

**Affiliations:** ^1^Department of Psychiatry, Institute of Mental Health, University of British Columbia, Vancouver, BC, Canada; ^2^BC Mental Health and Substance Use Services Research Institute, Provincial Health Services Authority (PHSA), Vancouver, BC, Canada

**Keywords:** impulsivity, decision-making, drug use, mental health, concurrent disorders, treatment outcome

## Abstract

**Background:**

Recent studies have employed computational modeling to characterize deficits in aspects of decision-making not otherwise detected using traditional behavioral task outcomes. While prospect utility-based modeling has shown to differentiate decision-making patterns between users of different drugs, its relevance in the context of treatment has yet to be examined. This study investigated model-based decision-making as it relates to treatment outcome in inpatients with co-occurring mental health and substance use disorders.

**Methods:**

50 patients (*M*age = 38.5, *SD* = 11.4; 16F) completed the Cambridge Gambling Task (CGT) within 2 weeks of admission (baseline) and 6 months into treatment (follow-up), and 50 controls (*M*age = 31.9, *SD* = 10.0; 25F) completed CGT under a single outpatient session. We evaluated 4 traditional CGT outputs and 5 decisional processes derived from the Cumulative Model. Psychiatric diagnoses and discharge data were retrieved from patient health records.

**Results:**

Groups were similar in age, sex, and premorbid IQ. Differences in years of education were included as covariates across all group comparisons. All patients had ≥1 mental health diagnosis, with 80% having >1 substance use disorder. On the CGT, patients showed greater Deliberation Time and Delay Aversion than controls. Estimated model parameters revealed higher Delayed Reward Discounting, and lower Probability Distortion and Loss Sensitivity in patients relative to controls. From baseline to follow-up, patients (*n* = 24) showed a decrease in model-derived Loss Sensitivity and Color Choice Bias. Lastly, poorer Quality of Decision-Making and Choice Consistency, and greater Color Choice Bias independently predicted higher likelihood of treatment dropout, while none were significant in relation to treatment length of stay.

**Conclusion:**

This is the first study to assess a computational model of decision-making in the context of treatment for concurrent disorders. Patients were more impulsive and slower to deliberate choice than controls. While both traditional and computational outcomes predicted treatment adherence in patients, findings suggest computational methods are able to capture treatment-sensitive aspects of decision-making not accessible via traditional methods. Further research is needed to confirm findings as well as investigate the relationship between model-based decision-making and post-treatment outcomes.

## Introduction

Psychiatric comorbidities are prevalent among substance users (1, 2), and their co-occurrence (or concurrent disorders) contribute substantially to the global disease burden (3). Individuals with concurrent disorders pose greater challenges to public healthcare systems than any psychiatric disorder alone, such as with more emergency service utilization and higher rates of psychiatric hospitalization (4). Moreover, treatment services are often ill-equipped to effectively manage the issues of mental health and substance use concurrently (1, 5, 6), and this could in part be attributed to the relatively few data representative of concurrent disorders patients as a coherent group in treatment (5). Research has historically focused on individual psychiatric disorders studied separately from one another (7), and drug use has frequently been treated as a criterion for participant exclusion from clinical study (5, 8). Given the heterogeneity of clinical characteristics with concurrent disorders that can vary vastly from persons to persons (9), multidisciplinary approaches aimed at addressing common underlying issues in the treatment are needed (10). While mental healthcare settings are increasingly adopting integrated care and showing it benefits to improved outcomes (11, 12), limited evidence supports the clinical management guidelines that have been mostly derived through studies of individual psychiatric disorders (5, 12). Thus, research representative of concurrent disorders patients, collectively as a single clinical group, are needed to better inform the development of interventions for broader spectrum problems and risks underlying poor treatment outcomes.

Suboptimal decision-making under conditions of risk or uncertainty [or risky decision-making; (13)] has been reported in individuals with schizophrenia (14), bipolar disorder (15), depression (16), anxiety (17), and various substances of use (18). Decision-making is often assessed using task-based measures of impulsivity. High levels of impulsivity and risk-taking are implicated in the development, maintenance, and severity of substance dependence (19, 20) and mental health disorders (21) and are associated with negative treatment outcomes, including poorer treatment adherence, higher rates of rehospitalization, morbidity, and mortality (22). Where problems in decision-making have been implicated in mental health disorders (23–25) and substance use disorders (19), impulsivity and risk-taking are also key risks where both psychiatric disorders co-occur (26–29). Recent evidence suggests individuals with co-occurring mental health and substance use disorders exhibit greater impulsivity than those with a single disorder (30). Studies have employed behavioral models, such as the Cambridge Gambling Task [CGT; (31)], to examine decision-making involving risk and reward. While robust evidence shows that deficits in CGT performance (19, 32, 33) predict adverse drug use outcomes (e.g., quality of decision-making, risk-taking), no studies to date have investigated its relevance in the context of treatment for concurrent disorders.

Recent advances with computational modeling have yielded techniques that can assess more nuanced aspects of decision-making that traditional outcomes have not been sensitive or capable of capturing (34). These model-based analyses can characterize subtle variation in cognitive processes underlying risk behavior, amending a significant limitation of traditional approaches in identifying underlying sources of behavioral task deficits (34). By more directly assessing cognitive processes underlying choice behavior, as opposed to overt task performance as a proxy for cognitive functioning, modeling may reduce interpretative bias and increase reproducibility of behavioral data, systematizing our understanding of cognition that underlies decision-making (35, 36). Moreover, the advantages to model-based approaches include their potential to detect aspects of cognition relevant to psychiatric diagnoses (37, 38). For example, the Prospect-Utility function has been applied to generate quantifiable parameters reflecting cognitive-motivational processes (e.g., reward valuation), based on an individual's choice patterns. Studies utilizing this approach with substance-using [e.g., (39, 40)], and other psychiatric populations (41) have identified distinct cognitive impairments underpinning choice behavior, as compared to controls (39) and between users of different drugs (42). While these computational methods show advantages over traditional methods in identifying specific cognitive indices of decision-making, they have yet to be studied in concurrent disorders and the clinical relevance of these computational data have yet to be explored (34, 37).

This study investigated risky decision-making in patients with concurrent disorders and assessed the utility of decision-making outcomes derived from computational modeling in predicting treatment outcomes. First, we hypothesized that patients would show worse decision-making performance than controls. Second, in patients, decision-making would predict treatment outcome. Third, the patterns of relationship between treatment outcome and decision-making would differ between indices of task performance collected through traditional techniques vs. those derived from computational modeling.

## Methods

### Participants

An initial 56 inpatient and 50 control males and females were recruited for a broader study investigating cognitive functioning and stress. Data from this broader study were not reported here, given they addressed a separate set of hypotheses. Participants had to be 19 years or older and fluent in English. They were excluded if they self-reported a history of neurologic disorder, or if they had uncorrected visual or auditory deficits.

Patients were recruited from the Burnaby Centre for Mental Health and Addictions, a 100-bed tertiary care facility. As required for treatment admission, all patients had to have co-occurring mental health and substance use disorders confirmed at intake by a licensed medical or mental health professional. Standard care included medications, individual and group psychotherapy (emphasizing harm reduction leading to abstinence), stepped care, and case management for up to 9 months [see (43)]. All patient participants were cleared by the unit psychiatrist, where patients had to be stable on medications and not exhibiting signs of withdrawal. Psychiatric diagnoses and discharge information (treatment length of stay, and reasons for discharge) were retrieved from patient medical records and reviewed by a PhD clinician.

Controls were volunteers recruited via community flyers and online advertisements. Self-reports and structured interviews probing medical history, mental health status, and drug use were administered by trained research staff. Controls could not have any current or chronic mental health disorder and/or a current or past substance use disorder.

Informed consent was obtained in accordance with procedures approved by the University of British Columbia Behavioral Research Ethics Board.

### Experimental Protocol

All eligible patients were invited to undergo two separate testing sessions, within the first two weeks of admission (baseline) and again 6 months into treatment (follow-up). Controls completed the same baseline assessments in a single outpatient session hosted at the university. Demographics, drug use [Addiction Severity Index–Lite, D1-D13; (44)], and premorbid IQ [NART; (45)] were assessed at baseline. At each session, participants were administered the Cambridge Gambling Task (CGT) followed by a package of self-report questionnaires. These self-reports were administered as part of the broader investigation and are not discussed here. Upon completion of each visit, patients were compensated a $10 Starbucks gift card and controls received $10 cash.

### Measures

#### Cambridge Gambling Task

**Figure 1:**
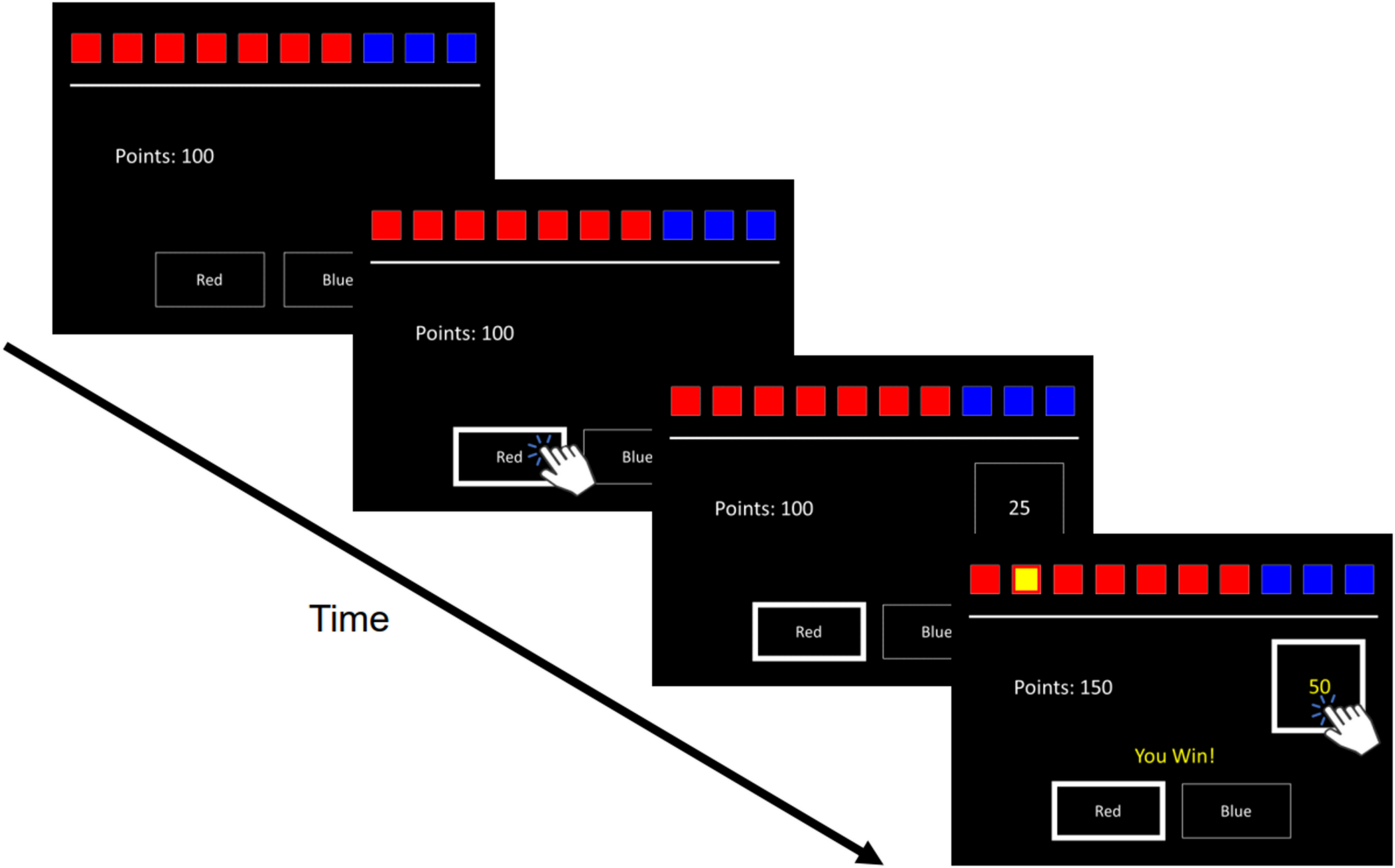
Trial Schematic of the Cambridge Gambling Task. Ten boxes are displayed varying in proportions of red to blue. Respondents guess which color hides a yellow token. Participants are then prompted to select a bet, with bet amounts appearing in either ascending or descending order. If respondents guess correctly or incorrectly, the selected bet amount is either added or subtracted to their total points.

The CGT is a standardized cognitive test used to assess decisions made under risk. On the screen are 10 boxes colored either red or blue (Figure 1). The ratio of red-to-blue boxes varied across trials. On each trial, participants had to guess whether a yellow token was hidden behind a red or blue box. With an initial endowment of 100 points, participants wagered points fixed to 5, 25, 50, 75, or 95% of their total standing points on having made the correct guess (blue or red box). Two within-subject task conditions presented betting options in either ascending (5, 25...95%) or descending (95, 75...5%) order. Because betting options were displayed one at a time with brief inter-interval delays, participants had to wait for their desired percentage bet to appear to place their bet. Instructions were to accumulate as many points as possible. Four traditional CGT outcomes were assessed: Quality of Decision-Making (QDM) is the proportion of trials participants chose the more likely color; Deliberation Time (DT) reflected the mean latency from the presentation of colored boxes to participants making a bet choice; Delay Aversion (DA) measures the difference in betting ratios across ascending and descending conditions, where large differences would indicate more impulsive betting; and, Risk-Taking (RT) reflected the mean proportion of accumulated points participants wagered on trials they chose the more likely color (i.e., the color with the highest proportion of boxes).

#### Computational Model

Computational modeling of trial-by-trial choice data followed Romeu et al. “Cumulative Model” [CM; (46)] and was executed in R using the hBayesDM package (47). Compared to other models, the CM has been shown to produce the best fit for the data and yield high predictive and convergent validity with standard CGT outcomes (46). While a brief overview of the CM is provided, we refer to Romeu et al. (46) for comprehensive mathematical specifications.

The CM assumes each box color and bet option has an expected utility (EU; or a “perceived advantage”) relative to all other options. The probability that a particular option will be chosen is derived from its EU; hence, the CM constructs per-trial probability estimates for all possible color and bet options. For instance, an option with the highest EU is one which is perceived to provide the largest reward and lowest risk of loss; thus, the CM would assign this option with the highest probability of being chosen.

The CM generates parameter estimates from choice data to capture four latent aspects of decision-making: Probability Distortion (α), Loss Sensitivity (ρ), Delayed Reward Discounting (β), and Choice Consistency (γ). Estimates for all parameters are computed per participant/group. To control for individual preference for red or blue boxes, Color Choice Bias (0 ≤ *c* ≤ 1) is included as an additional fifth parameter, with values closer to 1 indicating red bias and values closer to 0 indicating blue bias.

Probability Distortion (0 ≤ α ≤ 5) is posited as the underlying mechanism driving Quality of Decision-Making. It describes the frequency at which individual's color choice aligns with the proportion of red-to-blue boxes displayed (“objective odds”). Objective probability weighting is captured by α = 1, with higher α values indicating more optimal choices.

Loss Sensitivity (0 ≤ ρ ≤ +∞) captures individual variation in sensitivity to loss vs. gain. A ρ <1 suggests decreased Loss Sensitivity (greater Risk-Taking), ρ > 1 suggests increased Loss Sensitivity (greater Loss Aversion), and ρ = 1 suggests there is no difference in sensitivity to loss vs. gain. Delayed Reward Discounting (where 0 ≤ β ≤ +∞), is the propensity for individuals to perceive rewards as less valuable the longer it takes to receive them. The CM assumes that the EU of a given bet option diminishes linearly with the passage of time, and β is the slope of this decline. Higher values for β suggests greater impulsivity and more rapid discounting over time (i.e., steeper slope). Choice Consistency (0 ≤ γ ≤ +∞) reflects the degree of randomness present in an individual's choices as compared to the model's predictions, where larger values indicate greater consistency and predictability of choice patterns.

### Statistical Analyses

Due to incomplete data and data loss, 4 controls did not have baseline computational outcomes, 2 patients were missing computational data for follow-up, and 1 was missing traditional data for follow-up. From the initial sample, data from 50 patients and 50 controls were included for final analyses.

Because the Hierarchical Bayesian Analysis (HBA) is not well adapted for within-group comparisons (48–50), a frequentist approach was employed for primary within- and between-group analyses. We report HBA posterior estimates of group-level means and difference distributions in our Supplementary Materials.

Demographics were compared between patients and controls using Chi-square and independent samples *t*-tests. All subsequent analyses controlled for demographics where group differences were indicated. Because outputs derived from the CM are assumed to be not normally distributed (46), rank analysis of covariance [ANCOVA; (51)] was used to identify group effects on CGT outcomes [e.g., (52)]. In patients, related-samples Wilcoxon Signed Rank test was performed on non-normal CGT outcomes data to test within-subject changes from baseline to 6-month follow-up. For treatment outcomes, discharge status was coded as a binary outcome variable (discharge against medical advice vs. planned treatment termination/completion). Logistic regressions were conducted on discharge status and linear regressions were performed on treatment length of stay, both with CGT outcomes as predictor variables.

To probe for potential sampling bias, Mann-Whitney U tests were performed to assess differences in baseline CGT outcomes between patients who did vs. did not complete the follow-up session. Moreover, Spearman's Rank order correlations were used to test potential influence of psychiatric diagnoses (mental health disorders, substance use disorders) on obtained results. Significant correlations were followed up with rank repeated-measures ANCOVA to reassess outcomes with diagnosis included as a covariate (53). Original results (without diagnosis) were reported if there were no differences in outcome between controlling vs. not controlling for diagnoses.

For parametric tests (regressions), isolated univariate outliers with z-scores > 3.29 were truncated to one increment higher or lower than the closest non-outlier value within that group (54). We reported results from original data if there were no difference in outcome from truncated data. Data for non-parametric tests were not treated for potential outliers, given rank-based tests are robust to outliers. All analyses were computed using SPSS version 27.0 (IBM, Armonk, NY).

## Results

### Participants

Demographic characteristics are depicted on Table 1. Patients had fewer years of education whereas similar estimated premorbid IQ and age relative to controls. Because of group differences on education, years of education was included as a covariate for all subsequent group comparisons. There were no differences by age or group composition of males vs. females.

**Table 1:** Demographic characteristics.

	**Patients**	**Controls**
*N*	50 (16*F*)	50 (25*F*)
Age	38.5 ± 11.4	31.9 ± 10.0
Education (years)	10.8 ± 2.8^*^	16.5 ± 2.9
Estimated premorbid IQ	103.2 ± 7.3	107.8 ± 9.5
**Race/Ethnicity**	***N*** **(%)**	***N*** **(%)**
White	35 (70)	20 (40)
Indigenous	10 (20)	1 (2)
Black	1 (2)	1 (2)
Asian	1 (2)	25 (50)
Latinx	1 (2)	2 (4)

*Data presented as means ± SD, except where otherwise specified*.

* *p < 0.05*.

In patients, rate of disorders by diagnostic categories were 46% psychotic, 46% mood, 26% anxiety and stress-related, and 8% attention-deficit/hyperactivity disorder (Table 2), and 80% of all patients had >1 substance use disorder. Self-reported lifetime illicit drug use of highest prevalence in patients were cocaine (60%), heroin (60%), and methamphetamine (56%), with use averaging 11.5, 7.5, and 7.4 years, respectively (Table 3). The three most common drugs recently used were cocaine (46%), opioids (24%), and sedatives/tranquilizers (24%). In controls, 2% reported cocaine, 2% polydrug, and 4% non-prescription amphetamine use in the past 30-days.

**Table 2:** Patient diagnoses.

	**Patients *N* (%)**
*N*	50
**Substance use disorders**
>1 disorder	40 (80)
Alcohol only	5 (10)
Methamphetamine only	1 (2)
Subthreshold	4 (8)
**Mental health disorders**
Psychotic disorders	23 (46)
Schizophrenia/schizoaffective/unspecified	12/3/8
Mood disorders	23 (46)
Bipolar/depressive/unspecified	8/8/7
Anxiety disorders	13 (26)
Social/generalized/unspecified	5/2/1
PTSD	5 (10)
ADHD	4 (8)

*ADHD, attention-deficit/hyperactivity disorder; PTSD, posttraumatic stress disorder*.

**Table 3:** Substance use in patients.

	**Patient**		**Patient *(cont'd)***
**Polydrug**		**Methamphetamine**	
Lifetime any use (*n*)	42	Lifetime any use (*n*)	28
age onset^a^, ^b^	16.0 ± 5.7	age onset^a^, ^b^	24.0 ± 10.6
years used^b^	15.8 ± 12.1	years used^b^	7.4 ± 7.3
Past 30-day user (*n*)	31	Past 30-day user (*n*)	10
days used^c^	14.8 ± 11.4	days used^c^	12.9 ± 9.7
**Alcohol**		**Heroin**	
Lifetime any use (*n*)	44	Lifetime any use (*n*)	30
age onset^a^, ^b^	11.9 ± 3.5	age onset^a^, ^b^	27.0 ± 10.0
years used^b^	19.8 ± 12.8	years used^b^	7.5 ± 10.5
Past 30-day user (*n*)	27 (57.0)	Past 30-day user (n)	11
days used^c^	19.8 ± 12.8	days used^c^	8.3 ± 9.7
**Cigarettes**		**Other opioids**	
Lifetime any use (*n*)	36	Lifetime any use (*n*)	22
age onset^a^, ^b^	14.1 ± 6.4	age onset^a^, ^b^	23.1 ± 9.6
years used^b^	22.9 ± 11.8	years used^b^	8.7 ± 11.0
Past 30-day user (n)	23	Past 30-day user (n)	12
less than 10/day^d^	65%	days used^c^	14.0 ± 12.8
11-20/day^d^	22%	**Sedatives/tranquilizers**	
21+/day^d^	13%	Lifetime any use (*n*)	21
**Cannabis**		age onset^a^, ^b^	19.4 ± 5.7
Lifetime any use (*n*)	43	years used^b^	7.7 ± 7.5
age onset^a^, ^b^	13.2 ± 3.0	Past 30-day user (*n*)	12
years used^b^	18.0 ± 13.7	days used^c^	19.5 ± 10.2
Past 30-day user (*n*)	25		
days used^c^	14.7 ± 11.3		
**Cocaine**
Lifetime intranasal/	3/10/30		
smoked/both (*n*)			
age onset^a^, ^b^	21.6 ± 7.7		
years used^b^	11.5 ± 10.0		
Past 30-day user (*n*)	23		
days used^c^	12.7 ± 10.7		

*Data presented are means ± SD, except otherwise specified. Number of days used in past 30 days underestimate average use per month due to overlap with days in treatment*.

a *Polydrug: n = 31, alcohol: n = 38, cigarettes: n = 35, cannabis: n = 35, cocaine n = 34, methamphetamine: n = 28, heroin: n = 23; sedatives/tranquilizers: n = 14, due to missing data*.

b *Data from patients who reported lifetime ≥1x use of the substance*.

c *Data from patients who reported past 30 days ≥1x use of the substance*.

d *Data based on responses on Fagerstrom Test for Nicotine Dependence [n = 23; (55)]*.

### Group Differences on CGT Measures

**Figure 2:**
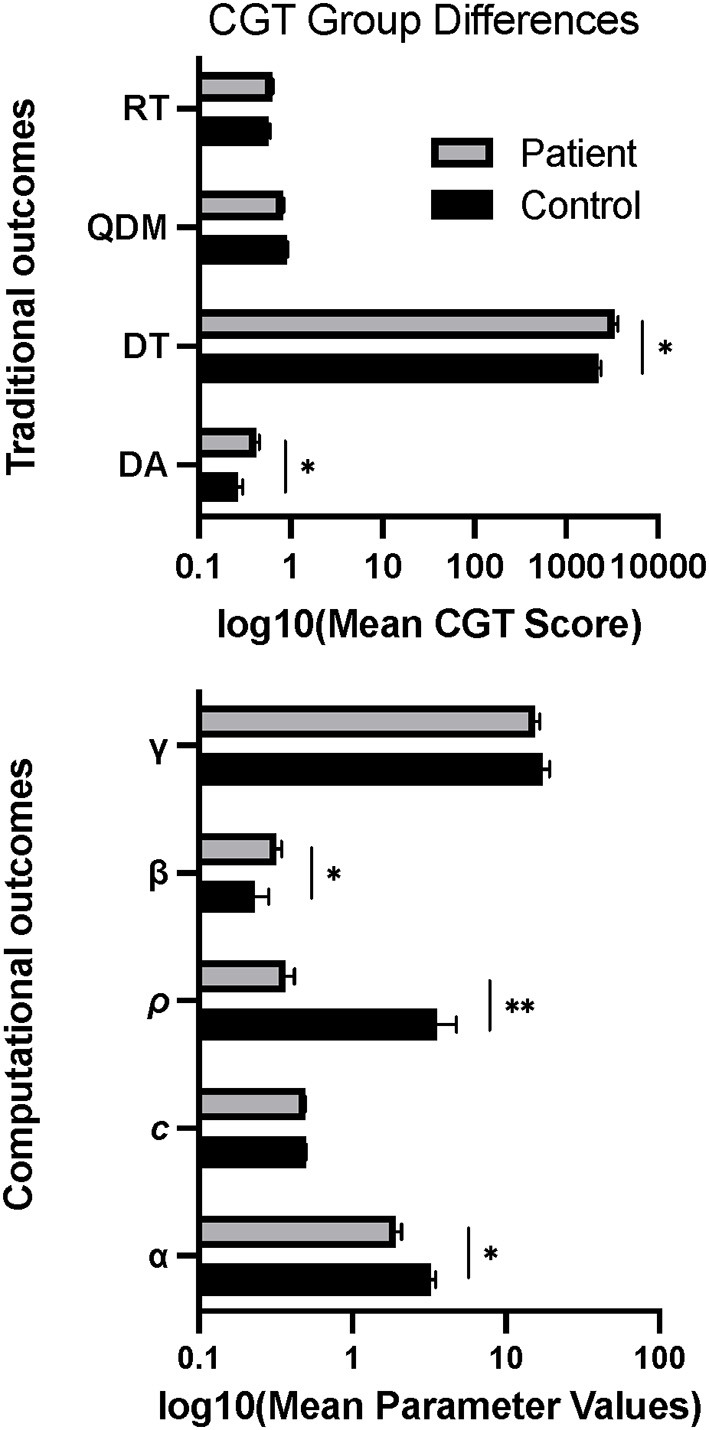
Group differences in mean scores on behavioral and computational CGT measures. Error bars show standard error. *p < 0.05, **p < 0.01.

On traditional CGT outcomes, patients exhibited higher Delay Aversion (*F*_(1, 98)_ = 12.96, *p* = 0.017) and Deliberation Time (*F*_(1, 98)_ = 22.01, *p* = 0.012) than controls (Figure 2). There were no group differences on Risk Taking or Quality of Decision-Making. For CGT model-based outcomes, patients exhibited lower Probability Distortion (*F*_(1, 94)_ = 20.54, *p* = 0.025), lower Loss Sensitivity (*F*_(1, 94)_ = 29.43, *p* = 0.007), and higher Delayed Reward Discounting (*F*_(1, 94)_ = 22.55, *p* = 0.013) relative to controls (Figure 2), with no group differences for Choice Consistency (γ) or Color Choice Bias (*c*).

**Figure 3:**
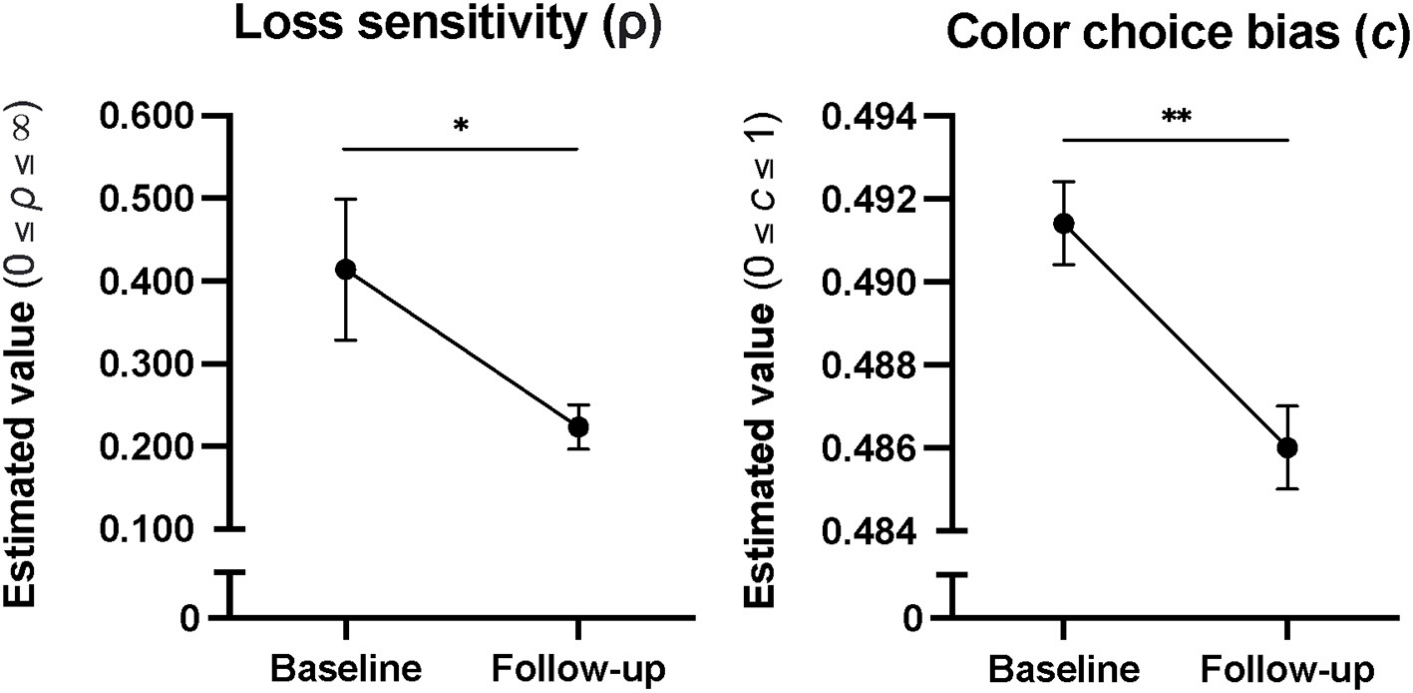
Cumulative model parameter estimates in patients from baseline to 6 months into treatment, showing decreases in Color Choice Bias and Loss Sensitivity over time. Error bars represent standard error. *p < 0.05, and **p < 0.01.

In patients (*n* = 24), there was a decrease in Loss Sensitivity (*d* = 0.36, *p* = 0.039) and Color Choice Bias (*d* = 0.01, *p* = 0.003) from baseline to 6-month follow-up (Figure 3). No other changes in CGT outcomes across time were statistically significant. Comparisons between patient follow-up completers and non-completers revealed higher baseline Delayed Reward Discounting in non-completers (Mann-Whitney Uc = 200.0; *p* = 0.031), with no other differences in baseline CGT outcomes. There were no significant correlations between psychiatric diagnoses and CGT outcomes on CGT differences scores (follow-up minus baseline) to warrant follow-up with diagnoses included as a covariate in the statistical model.

### CGT Performance and Treatment Outcome in Patients

There was a significant relationship between baseline CGT performance and treatment outcome, such that higher Quality of Decision-Making (OR = 168.17, *p* = 0.032) and Choice Consistency (OR = 1.08, *p* = 0.045) and lower Color Choice Bias (or greater blue bias; OR = 0.00, *p* = 0.027) predicted greater likelihood of adherence to treatment (Table 4). No other traditional or computational CGT outcomes were associated with reason for discharge, and there was no relationship between CGT outcomes and treatment length of stay.

**Table 4:** Logistic regression predicting treatment outcome (unplanned vs. planned).

						**95% CI for OR**	
**Variable**	**B**	**SE**	**Wald**	**df**	**OR**	**Lower**	**Upper**
DT	0.00	0.00	1.63	1	1.00	0.99	1.00
DA	0.28	1.34	0.04	1	1.32	0.10	18.05
QDM^*^	5.13	2.39	4.59	1	168.17	1.55	18295.14
RT	3.35	2.56	1.71	1	28.41	0.19	4302.72
α	0.37	0.24	2.49	1	1.45	0.91	2.30
ρ	−0.94	0.81	1.33	1	0.39	0.08	1.93
β	0.55	1.43	0.15	1	1.73	0.11	28.44
γ^*^	0.08	0.04	4.00	1	1.08	1.00	1.16
*c* ^*^	−205.89	93.31	4.87	1	0.00	0.00	0.00

*α, Probability Distortion; c, Color Choice Bias; ρ, Loss Sensitivity; β, Delayed Reward Discounting, γ, Choice Consistency; CI, confidence interval; DA, Delay Aversion; DT, Deliberation Time; OR, odds ratio; RT, Risk Taking; SE, standard error; QDM, Quality of Decision-Making*.

* *p < 0.05*.

## Discussion

Our study extends findings of computational modeling of decision-making under risk to concurrent disorder in-patients and is the first to characterize the relationship between treatment outcome and decisional processes in this population. On traditional CGT measures, patients had longer deliberation times and greater delay aversion at baseline than controls. With model-based CGT outcomes, patients showed lower loss sensitivity and probability distortion, and higher delayed reward discounting relative to controls. In patients, while aspects of decision-making elucidated using model parameters showed decreases in (red) color choice bias and loss sensitivity from baseline to 6 months into treatment, no changes were indicated for measurements of traditional indices of CGT performance. Moreover, some behavioral aspects of decision-making, as assessed via both traditional and computational methods, were found to predict treatment outcome. Higher choice consistency and quality of decision-making, and lower (red) color choice bias at baseline was associated with greater likelihood of treatment adherence, with no observed relationship between decision-making and treatment length of stay.

In line with prior research, patients performed worse on CGT task outcomes, as measured by traditional and computational methods, with the model-based approach revealing group-differences undetected by traditional measures of behavioral performance. Data showing greater baseline deliberation time and delay aversion in patients are consistent with previous reports of psychomotor slowing and slower choice processing (56, 57), and aversion to delays (58) among patients with substance use disorders. Moreover, between-group patterns for model parameters were consistent with those reported in Romeu et al. (46), with substance use disorder patients exhibiting similarly low loss sensitivity and probability distortion, and high delayed reward discounting estimates relative to healthy controls.

Lower loss sensitivity, as conceptualized by Romeu et al. (46), is indicative of riskier behavior. However, decrease in loss sensitivity as it relates to treatment in our study is not well understood. Further research is needed to elucidate the functional and clinical significance of cognitive processes that are malleable with treatment, including their relation to post-treatment outcomes (e.g., mortality, relapse, symptom reduction, drug use). Nonetheless, these findings demonstrate the clinical relevance of this aspect of decision-making and support further investigation of decisional processes as they relate to post-treatment outcomes.

Comparisons between patients who completed follow-up vs. those who did not complete follow-up revealed greater baseline delayed reward discounting among non-completers, with no other differences. Delayed reward discounting is a well-established risk for poor treatment outcomes among substance users, including treatment adherence [for review, see (59)]. Hence, non-completers may have been more likely to have prematurely terminated their treatment, however, our examination of the patient data did not reveal this to be the case. Given there were no other differences between patient completers and non-completers, results for decreased loss sensitivity and color choice bias across time in treatment were unlikely to have been influenced by sample bias.

Lower quality of decision-making and choice consistency, and higher values for color choice bias at baseline were identified as predictors for greater likelihood of unplanned treatment termination (against medical advice). These findings are consistent with prior research reporting the negative influence of inconsistent choice bias (60) and suboptimal quality of decision-making [i.e., rational choice passed on probability; (32)] in lowering the rate of treatment retention among substance use disorder patients. Color choice bias emerged as findings consistent with those reported in the prior study (46). Although originally formulated as a control for noise in CM, some evidence suggests a blue color choice bias may be associated with drug-related dopaminergic activity (61–63) and drug use status (63). Future investigations may further examine for a possible link between perceptual color bias and drug use, and their influence on choice patterns in mental health and substance-using populations. Likewise, the absence of change in behavioral indices of decision-making performance, specifically those that predicted poorer treatment outcomes, also highlight potential areas to examine novel targeted interventions.

While the heterogeneity of individuals and patient samples with concurrent disorders can be vast (9), computational approaches offer an opportunity to further advance our understanding of the potential common denominators (e.g., constituent processes in decision-making) leading to poor treatment outcomes in the broader clinical groups as a whole. Model-based assays for cognitive factors underpinning symptoms and disorders may yield insights into concurrent disorders and potential treatments, especially since psychopathology beyond symptom count have been understudied in this population (12).

It is increasingly clear that transdiagnostic risk factors contribute to mental disorders, be they substance induced or not. In order to better understand and treat multimorbidity such as concurrent disorders further development of computational models are needed.

This study includes notable limitations. First, attention deficit hyperactivity disorder (ADHD) was not excluded in the sample. Because individuals with ADHD receive treatment in psychiatric care settings, and they tend to have high rates of comorbidity with co-occurring mental health conditions (64), the inclusion of this disorder was consistent with the study's main objectives in assessing a representative sample of patients with concurrent disorders as a group. Second, patients had a low rate of attendance for testing at 6-month follow-up. Non-completers may have been overall more severe patients, as this was supported by worse delayed discounting at baseline relative to completers. Because we found no differential baseline performances on measures that changed during treatment, it is unlikely longitudinal findings were due to sample bias. Third, findings cannot speak to sex differences. Our sample did not comprise enough females to perform subgroup comparisons. Future assessment of sex and gender differences is warranted. Fourth, given the cross-sectional, non-experimental design, the etiology of differences in decision-making cannot be determined. However, with evidence to demonstrate alteration in aspects of decision-making in response to treatment. Regardless of causality, the clinical implications are important and need to be further explored, including broader longitudinal follow-ups investigating their relevance in relation to outcomes post-treatment (e.g., relapse, overdose, rehospitalization). Fifth, given the heterogeneity of the concurrent disorder population, factors such as psychiatric disorders and their related clinical characteristics may have driven some results more than others, including those derived through modeling. However, statistical controls for diagnoses were carefully tested to confirm this to be unlikely. Further, there is currently little evidence to suggest there are substance-specific effects on decision-making, and part of the reason is precisely because of the complication of overlapping drug use across many types of different drugs (65). Alternatively, distinguishing outcomes by diagnoses was beyond the scope of the study. Lastly, a primary limitation of computational models is that they do not have explanatory power of the cognitive phenomena underlying behavior but rather, form quantitative predictions of how behavior is generated (34, 66). More research is needed to establish generalizability of parameter interpretations and to incorporate other factors that may be of importance to choice processes, such as affective state and environmental factors (34, 67). The purpose was to study concurrent disorders patients as a single coherent group and to examine the clinical utility of computationally modeled decision-making behaviors.

Limitations notwithstanding, this is the first study to demonstrate clinical utility of decision-making in concurrent disorders populations. Our results underscore the advantages of computational models in assessing functional impairment in psychiatric disorders, as compared to traditional approaches. Findings support further investigation of model-based assessments of decision-making behaviors as they relate to mental health and substance use outcomes.

## Data Availability Statement

The original contributions presented in the study are included in the article/Supplementary Files, further inquiries can be directed to the corresponding author/s.

## Ethics Statement

The studies involving human participants were reviewed and approved by University of British Columbia Behavioral Research Ethics Board. The patients/participants provided their written informed consent to participate in this study.

## Author Contributions

ST conducted data analyses and drafted the introduction, results, discussion, and figures. LS computed all data used in the computational analyses. LS and KT wrote the methods section, drafted the tables, and contributed to revising the overall manuscript. TC and CS supervised ST in the data analytic plan, drafting of the manuscript, and revised the manuscript for important intellectual content. All authors contributed to the article and approved the submitted version.

## Funding

This work was supported by Institution funding from the Provincial Health Services Authority, British Columbia, Canada.

## Conflict of Interest

The authors declare that the research was conducted in the absence of any commercial or financial relationships that could be construed as a potential conflict of interest.

## Publisher's Note

All claims expressed in this article are solely those of the authors and do not necessarily represent those of their affiliated organizations, or those of the publisher, the editors and the reviewers. Any product that may be evaluated in this article, or claim that may be made by its manufacturer, is not guaranteed or endorsed by the publisher.
