# Supplementary material for: Changes in Loss Sensitivity During Treatment in Concurrent Disorders Inpatients: A Computational Model Approach to Assessing Risky Decision-Making
Source: Front Psychiatry. 2022 Jan 28;12:794014. doi: 10.3389/fpsyt.2021.794014 (PMC8831914; doi:10.3389/fpsyt.2021.794014)
Supplement: Supplementary file 1 [file Data_Sheet_1.docx]

**Supplementary Materials**

**Changes in Loss Sensitivity During Treatment in Concurrent Disorders Inpatients: A Computational Model Approach to Assessing Risky Decision-Making**

Stefanie Todesco^1^, Thomas Chao^1^, Laura Schmid^1^, Karina A. Thiessen^1^, Christian G. Schütz^1,2*^

^1^Institute of Mental Health, Department of Psychiatry, University of British Columbia, BC, Canada

^2^BC Mental Health & Substance Use Services Research Institute, PHSA, BC, Canada

* Correspondence: Christian G. Schütz MD, PhD, MPH, FRCPC, Institute of Mental Health, Department of Psychiatry. 5950 University Boulevard, Vancouver, BC V6T 1Z3, Canada. Phone: +1 778 873 4785. Email: christian.schutz@ubc.ca.

**1. Methods**

*Statistical Analyses*

We generated CM group-level posterior estimates at baseline for healthy controls and patients and posterior distributions of group mean differences parameters (see Romeu et al., 2020). Evidence of a strong likelihood for group differences were represented as 95% highest density intervals (HDI) that excluded (or nearly excluded) a parameter difference value of 0 (Kruschke, 2014).

**2. Results**

Group level posterior estimates for patients and controls at baseline are depicted in Figure S1. We found strong evidence for differences across groups such that patients displayed lower Probability Distortion (α), higher Delayed Reward Discounting (β), and lower Loss Sensitivity (ρ) than controls (Figure S2). These results are consistent with our main findings derived from non-parametric tests and similar to those reported in Romeu et al. (2020).

**
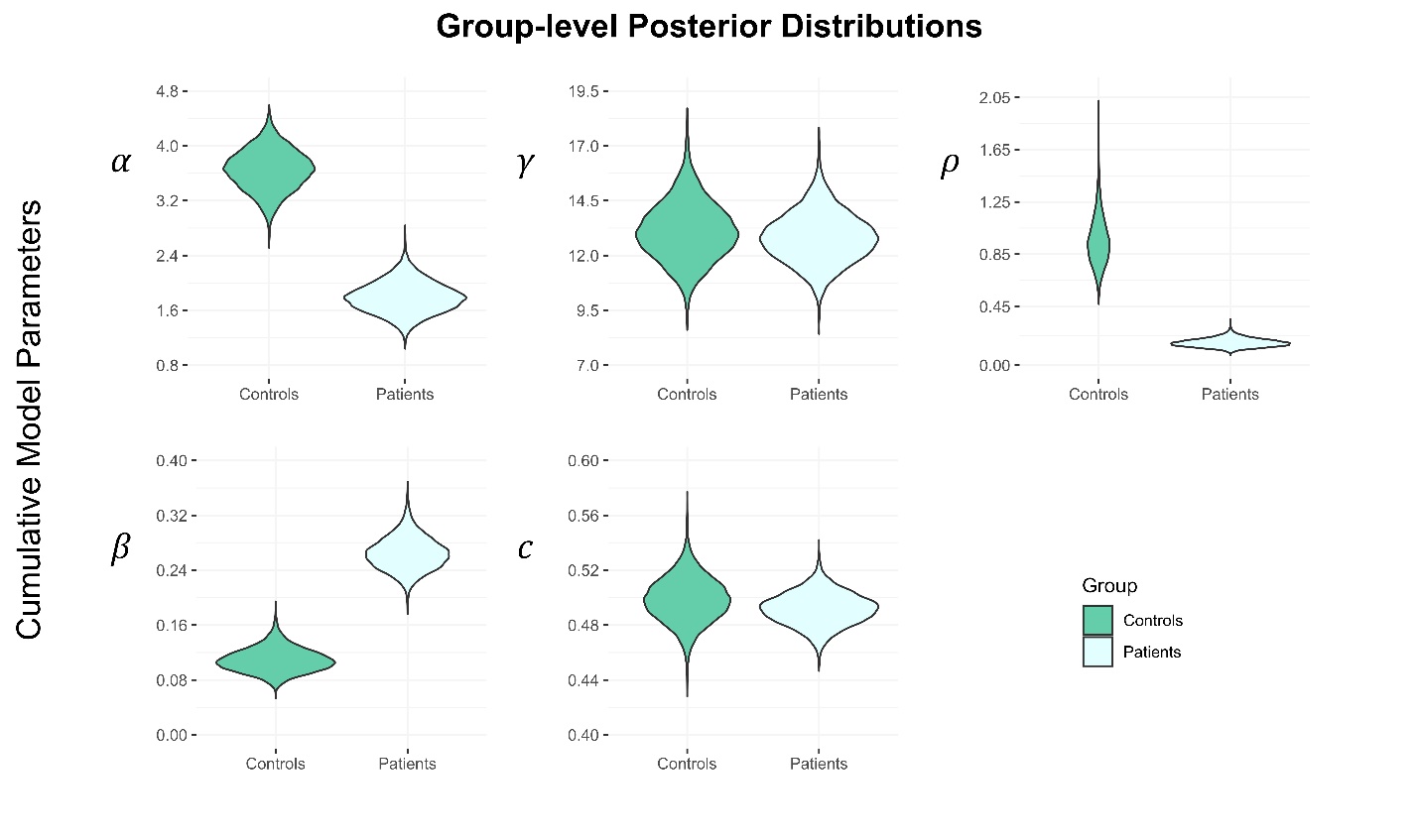
**

**Figure S1.** Group-level posterior estimates of parameters from the Cumulative Model.

**
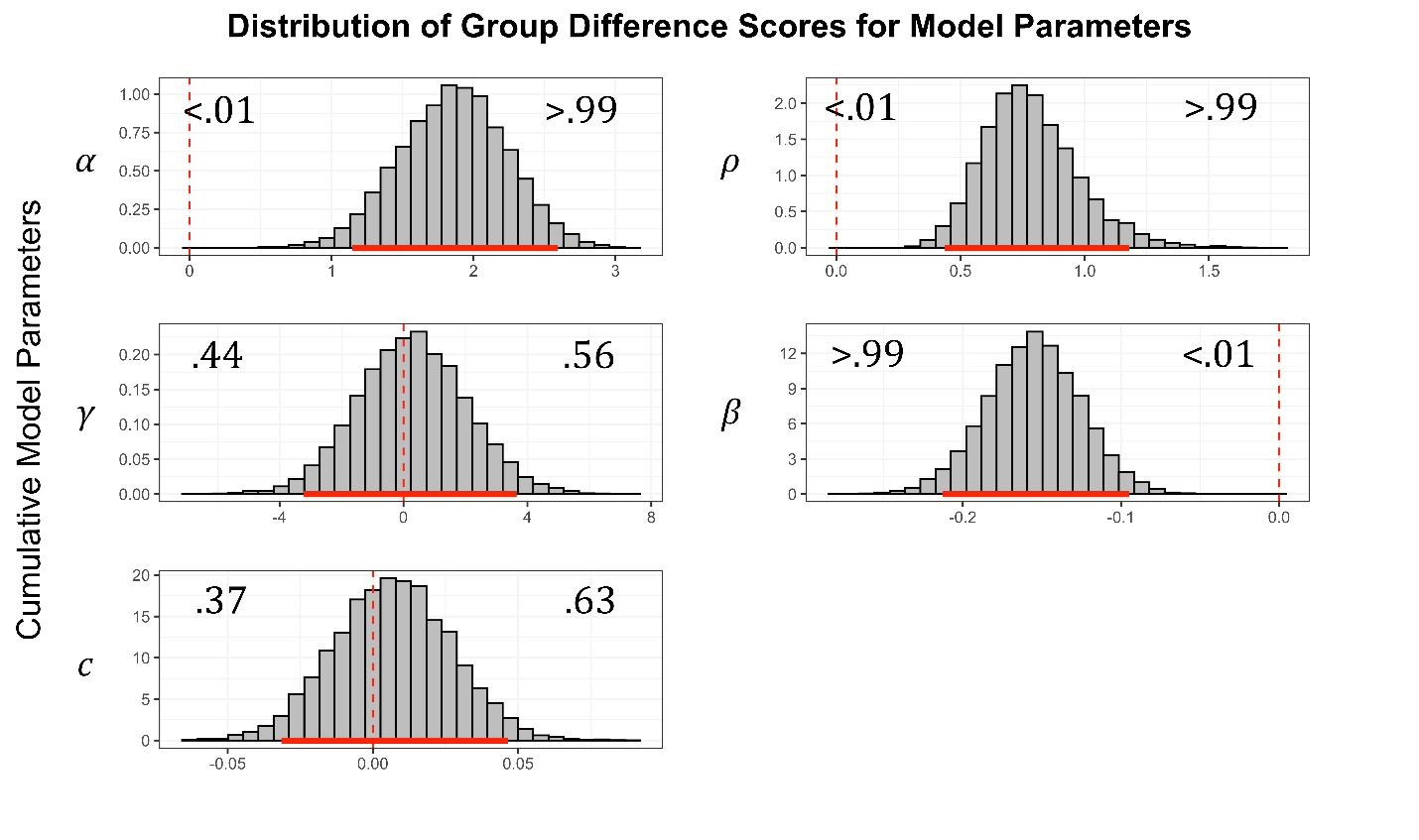
**

**Figure S2.** Posterior distribution of parameter estimated differences between groups. Differences were computed as the mean of a parameter in controls minus that in patients. The red underline reflects the 95% Highest Density Interval (HDI) for each group difference and dotted line is where there is zero difference between groups. Data shows a strong likelihood Patients were lower in Probability Distortion, higher in Delayed Reward Discounting, and lower in Loss Sensitivity than controls.

References

Kruschke, J. (2014). Doing Bayesian data analysis: A tutorial with R, JAGS, and Stan.

Romeu, R.J., Haines, N., Ahn, W.-Y., Busemeyer, J.R., and Vassileva, J. (2020). A computational model of the Cambridge gambling task with applications to substance use disorders. *Drug and Alcohol Dependence* 206**,** 107711.
